# Ultrasound-Guided Percutaneous Nerve Stimulation in Post-Stroke Spasticity: A Case Report

**DOI:** 10.3390/neurolint17030034

**Published:** 2025-02-24

**Authors:** Francesco Sartori, Albert Puig-Diví, Javier Picañol

**Affiliations:** 1 School of Health Sciences Blanquerna, Ramon Llull University, 08022 Barcelona, Spain; 2 Laboratory of Neurophysiology, Biomedicine Department, Faculty of Medicine and Health Sciences, Institute of Neurosciences, University of Barcelona, 08036 Barcelona, Spain; 3 Department of Health Sciences, TecnoCampus University Center, Pompeu Fabra University, 08302 Barcelona, Spain

**Keywords:** percutaneous peripheral nerve stimulation, post-stroke spasticity, electrotherapy, neurological rehabilitation, case report

## Abstract

Introduction: Post-stroke spasticity (PSS) significantly impacts the quality of life for stroke survivors. While various treatments exist, options for refractory cases are limited. Ultrasound-guided percutaneous peripheral nerve stimulation (pPNS), commonly used in pain management, has not been studied for its potential use in spasticity management. This case report aims to evaluate the sensorimotor effects of pPNS in a patient with severe PSS. Case description: A 38-year-old male with severe PSS and functional limitations post-ischemic stroke in the middle cerebral artery underwent a six-week pPNS protocol (12 sessions). Low-frequency (2 Hz) stimulation targeted the median, musculocutaneous, and anterior interosseous nerves, while medium-frequency (10 Hz) stimulation targeted the posterior interosseous and radial nerves. Spasticity was assessed using the Modified Ashworth Scale (MAS) and Tardieu Scale (TS). Somatosensory assessments included tactile thresholds, pressure pain thresholds, and conditioned pain modulation (CPM). Outcomes: Spasticity decreased significantly, with reductions of 60.4% and 67.0% in elbow and wrist MAS scores, respectively, and a 49.5% reduction in TS scores. However, spasticity levels returned to baseline between sessions. Somatosensory assessments revealed increased tactile thresholds, decreased pressure pain thresholds, and an 81.3% reduction in CPM. The intervention was well tolerated, with minor transient effects, and the patient preferred pPNS over botulinum toxin injections. Conclusions: pPNS may effectively reduce spasticity and modulate somatosensory thresholds in PSS. These preliminary findings highlight its potential as an alternative treatment for refractory PSS, warranting further research with larger sample sizes and control groups to assess its broader clinical applicability.

## 1. Introduction

Stroke is one of the leading causes of death globally, but advances in healthcare have resulted in a high survival rate, making it one of the primary causes of disability. More than 80 million people worldwide have survived a cerebrovascular accident [1]. Stroke frequently leads to spasticity, with an incidence ranging from 4% to 42.6% [2]. Post-stroke spasticity (PSS) may arise from upper motor neuron damage that causes intermittent or sustained involuntary muscle contraction. If severe, it can negatively impact functionality and overall quality of life [3].

Treatment may be required to alleviate discomfort and stroke-related pain, improving patient function and participation in daily activities [3]. However, not all cases of PSS require intervention, as spasticity can sometimes be functionally beneficial [4]. When necessary, treatment can prevent complications. The most common approach combines pharmacological and non-pharmacological therapies. Medications such as oral baclofen or benzodiazepines target GABA receptors [5,6], while injectable treatments such as botulinum toxin inhibit the release of acetylcholine at the neuromuscular junction [7]. On the non-pharmacological side, physical therapy offers a variety of interventions [8]. Electrical stimulation presents itself as a possible approach to managing spasticity [9]. Evidence supports its ability to reduce spastic tone and improve motor function. It is hypothesized to modulate neuronal excitability and spinal reflex hyperactivity, potentially inducing spinal plasticity through increased presynaptic inhibition of motoneurons and enhanced reciprocal inhibition [10]. However, research has focused mainly on transcutaneous stimulation techniques such as TENS and NMES. In other fields, such as pain management, percutaneous peripheral nerve stimulation (pPNS) has emerged as an electrical stimulation technique to improve pain and function in various contexts [11,12,13]. This method involves ultrasound-guided needling to target the specific nerve, allowing the stimulation of nerve axons.

However, despite its potential, the application of this technique in spasticity management remains quite limited. This study presents a case report that examines the individual effects of pPNS on both spasticity and somatosensory function in a patient with moderate to severe PSS who did not respond to standard botulinum toxin treatment, which is typically considered a gold standard intervention for spasticity management [14]. To the best of our knowledge, this is one of the few documented cases exploring the use of pPNS in this specific patient profile, highlighting a potentially therapeutic option for refractory cases of PSS.

## 2. Case Description

A 38-year-old male, born in 1986, was admitted on 29 September 2023, for evaluation and management of persistent post-stroke spasticity and functional impairments. He had a history of a subacute ischemic stroke affecting the territory of the middle cerebral artery, which had occurred in 2020. The stroke was characterized by thrombosis of the right common carotid artery, extending to the middle cerebral artery and the A1 segment, the proximal portion of the anterior cerebral artery. Collateral circulation through the anterior communicating artery ensured partial perfusion to affected regions. Key medical examinations conducted at the time of the stroke revealed critical findings. On 3 October 2020, cranial CT angiography showed that the right A2 segment, the distal portion of the anterior cerebral artery, was perfused through the anterior communicating artery. Uncal herniation was observed on the right side, with a displacement of one cm from the right posterior cerebral artery. Imaging also revealed an infarct that encompassed the entire territory of the right middle cerebral artery. On the same day, a non-contrast cranial CT scan identified a subacute infarct with luxury perfusion (state of increased blood flow in an infarcted region) in the territory of the right middle cerebral artery. This infarct resulted in compression of the right lateral ventricle, subfalcine herniation, and right uncal herniation. Subfalcine herniation refers to the displacement of the cingulate gyrus beneath the falx cerebri, potentially causing compression of the anterior cerebral artery and contralateral motor deficits. Right uncal herniation describes the downward displacement of the medial temporal lobe through the tentorial notch, which can lead to oculomotor nerve compression, midbrain dysfunction, and visual or consciousness disturbances.

The patient had a genetic predisposition to cardiovascular events, as evidenced by a history of myocardial infarction in his father and ischemic stroke in his paternal grandmother, both unrelated to the prothrombin gene. Additional relevant comorbidities included optic neuritis and moderate obesity. Psychologically, the patient reported chronic fatigue, attention difficulties, and, as a computer engineer, chronic challenges in performing his work at the usual quality level. In the first month after stroke, he completed 70 in-home physical rehabilitation sessions focused on motor and sensory recovery, followed by more than 40 outpatient sessions, including mirror therapy and robotic-assisted therapy for motor function. Psychologically, he identified the first year as the most challenging for daily activities, with the stroke partially affecting his vision. Basic activities, such as personal hygiene and ambulation, were significantly affected. The patient also expressed a strong desire to address spasticity in his affected upper limb to improve both functionality and appearance. Due to the significant impact of post-stroke spasticity on his functional ability and participation in daily activities, the patient received botulinum toxin injections in the upper limb on 14 October 2020, to reduce spasticity, with electromyography (EMG) guidance. Despite this intervention, he reported minimal improvement in spasticity reduction and quality of life, and the lack of notable progress caused considerable distress. He also noted muscle atrophy in the injected areas, a documented side effect in previous studies [15]. The patient’s rehabilitation trajectory up to the date of the study included a botulinum toxin injection in the upper limb (October 2020), which was administered as part of a combined treatment approach that also included conventional rehabilitation in outpatient and home settings (October 2020–December 2021); robotic rehabilitation (January 2022–September 2022); and private neurorehabilitation therapy (October 2022–January 2023).

Despite ongoing PSS treatment, the patient continued to exhibit severe motor impairments and functional limitations. Spasticity assessments using the Modified Ashworth Scale (MAS) and the Modified Tardieu Scale (MTS) showed high muscle stiffness and resistance throughout the hemiparetic side. In addition, the patient experienced a reduced range of motion and significant difficulty in activities of daily living. The planned intervention involved ultrasound-guided percutaneous electrical stimulation, with the aim of improving motor control and reducing spasticity.

## 3. Intervention

**Figure 1:**
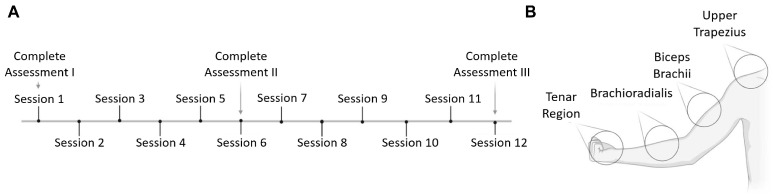
(**A**) Diagram of the case report, comprising 12 intervention sessions. Spasticity changes were assessed at each session, while comprehensive somatosensory evaluations were conducted at baseline, midpoint, and study completion. (**B**) Primary regions of the upper limb where somatosensory assessments of pressure pain thresholds and tactile thresholds were conducted. This figure was generated using BioRender.com (Version 1.0.0.3) under a licensed agreement.

The case study was conducted in accordance with international guidelines, complied with the ethical standards set forth in the Declaration of Helsinki [16], and has met institutional requirements of the Health Insurance Portability and Accountability Act (HIPAA) policy for the disclosure of protected health information. The intervention was structured as a continuous treatment, comprising 12 sessions in total over six weeks (Figure 1A), with two pPNS sessions per week. In each session, spasticity evaluations were performed before and after the intervention, and in Sessions 1, 6, and 12, a comprehensive evaluation was performed, including upper-limb somatosensory evaluations in multiple regions (Figure 1B). The protocol was thoroughly explained to the patient, ensuring informed consent prior to participation. The study adhered to the CARE guidelines for case reports [17].

**Figure 2:**
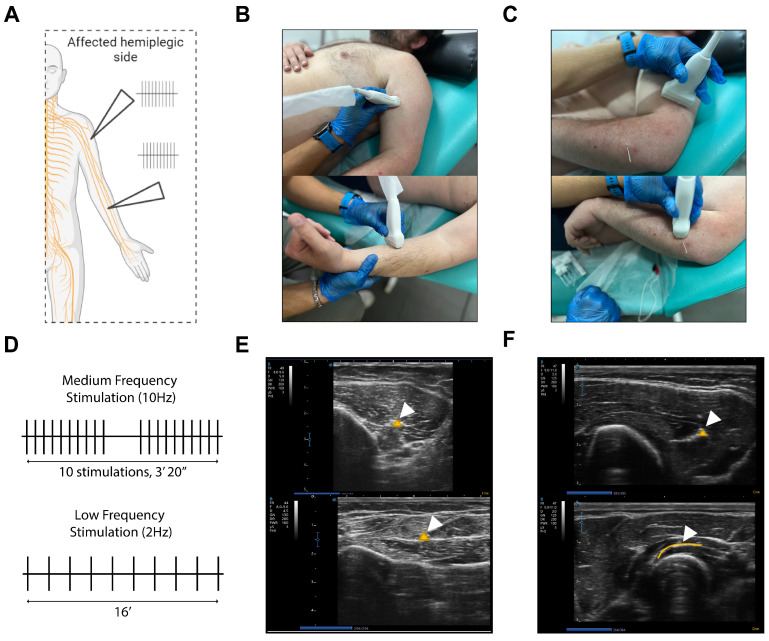
(**A**) Graphical representation of the ultrasound-guided intervention on the affected upper limb. (**B**) Probe positioning for ultrasound guidance of the intervention targeting the musculocutaneous nerve (upper panel) and the median nerve (lower panel). (**C**) Probe positioning for ultrasound guidance of the intervention targeting the radial nerve (upper panel) and the posterior interosseous nerve (lower panel). (**D**) Diagram of the interventions performed, illustrating a medium-frequency protocol and a low-frequency protocol. (**E**) Upper panel: Ultrasound visualization of the musculocutaneous nerve. Lower panel: Ultrasound visualization of the median nerve. (**F**) Upper panel: Ultrasound visualization of the radial nerve. Lower panel: Ultrasound visualization of the posterior interosseous nerve. In (**E**,**F**), pattern nerves are indicated by white arrowheads. Panel A was generated using BioRender.com under a licensed agreement.

The pPNS intervention involved ultrasound-guided puncture of various peripheral nerves in the affected limb (Figure 2A), divided into two distinct phases: (1) low-frequency stimulation at 2 Hz (Figure 2D, inferior panel) applied to the median, musculocutaneous, and anterior interosseous nerves for 16 min (Figure 2B,C) [18], followed by medium-frequency stimulation (10 Hz) targeting the posterior interosseous and radial nerves (Figure 2C,E). The medium-frequency protocol was based on previously described interventions [13,19], consisting of 10 pulse trains, each lasting 10 s, with 10-s rest intervals (Figure 2D, superior panel). The intensity of the electrical stimulation was adjusted according to patient tolerance to induce nonpainful involuntary contractions. The ITO130 (ITO Co., Ltd., Tokyo, Japan) device was used to deliver an asymmetric biphasic square wave with a pulse duration of 100 ms, while the ultrasound device (SONOSCAPE E2; SonoScape Medical Corp., Shenzhen, China) provided guidance for the intervention. Throughout the procedure, the affected limb was kept in a natural position to prevent posturing.

## 4. Outcomes

### 4.1. Spasticity Assessment

**Figure 3:**
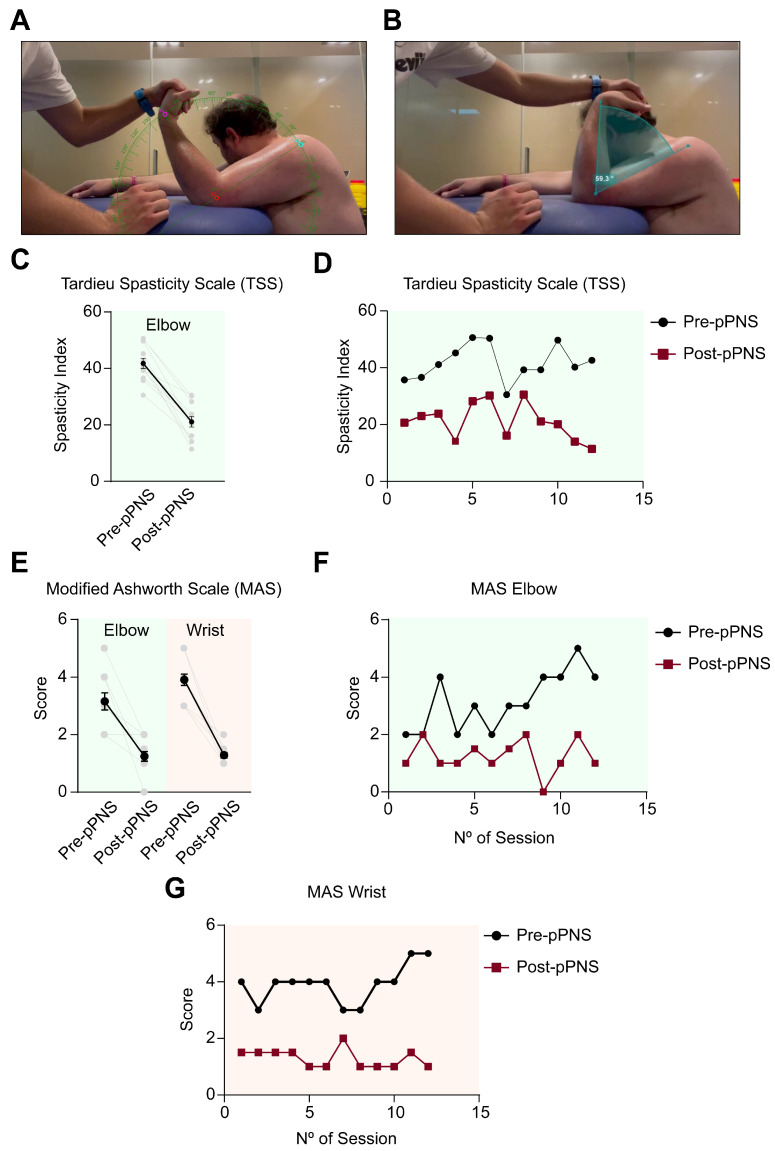
(**A**) Manual assessment of spasticity in the upper limb through passive mobilization to obtain Tardieu Spasticity Scale (TSS) scores. (**B**) Illustration of passive mobilization of the upper limb with ultrasound guidance for precise intervention positioning. (**C**) Comparative analysis of the spasticity index at the elbow using the Tardieu Spasticity Scale (TSS) before and after percutaneous peripheral nerve stimulation (pPNS). (**D**) Longitudinal changes in the spasticity index at the elbow across 12 intervention sessions, based on TSS measurements, contrasting pre-pPNS (black circles) and post-pPNS (blue squares) scores. (**E**) Comparative analysis of the Modified Ashworth Scale (MAS) scores for the elbow and wrist pre- and post-pPNS intervention. (**F**) Temporal progression of the MAS scores for the elbow across 12 sessions, comparing pre-pPNS (black circles) and post-pPNS (blue squares) results. (**G**) Temporal progression of the MAS scores for the wrist across 12 sessions, comparing pre-pPNS (black circles) and post-pPNS (red squares) results.

An experienced clinician assessed spasticity five minutes before and after the intervention using the Modified Ashworth Scale (MAS) across 12 sessions [20,21,22]. The Modified Ashworth Scale (MAS) assessment was conducted following a standardized protocol designed to enhance reliability and minimize variability across sessions. The patient was seated with the shoulder flexed at 90° and the elbow supported on a stable surface to ensure consistent positioning throughout the evaluation (Figure 3A). To reduce distractions and facilitate uniform testing conditions, the patient was instructed to maintain a neutral posture, close their eyes, and turn their head in the opposite direction during the assessment. For reproducibility, all evaluations were video-recorded using a high-definition camera positioned 2 m away from the patient, capturing the entire assessment field (Figure 3A). Anatomical landmarks—specifically, the greater tubercle of the humerus, the lateral epicondyle of the elbow, and the radial styloid process—were marked using a skin-safe marker to ensure precise and consistent angle measurement (Figure 3B). The MAS test was performed at a controlled and uniform speed, with three repetitions for each joint (elbow and wrist) both before and after the intervention. To enhance the precision of the evaluation, the video recordings were analyzed post-session using Kinovea software. This software facilitated the accurate measurement of joint angles and provided additional quantitative data, such as acceleration metrics, which complemented the examiner’s clinical judgment and helped validate the scoring process. To further ensure reliability and mitigate subjectivity, a second evaluator independently reviewed the video recordings. This secondary review included cross-referencing clinical observations with the software-derived metrics. This dual-evaluator approach provided an objective framework for assessing spasticity and improved the reproducibility and accuracy of the MAS scoring system.

The pPNS intervention led to a reduction in spasticity, as measured by the MAS, in both the elbow and wrist joints (Figure 3E). Spasticity in the elbow decreased from 3.16 ± 1.03 (CI95%: 2.51–3.82) to 1.25 ± 0.58 (CI95%: 0.87–1.62), reflecting a reduction of 60.4%, while in the wrist, it decreased from 3.91 ± 0.66 (CI95%: 3.49–4.34) to 1.29 ± 0.33 (CI95%: 1.07–1.5) (67.01% reduction). However, despite immediate post-intervention reduction, no sustained decrease in spasticity was observed prior to the intervention across the 12 sessions at either joint of the upper limb (Figure 3F,G). Following the MAS, the Tardieu Scale (TS) was administered under the same conditions [23]. Two key angles were measured to quantify spasticity: (1) R1, the total passive range of motion (ROM) in elbow extension, measured at a slow speed; and (2) R2, the “catch” angle, measured at a fast speed. The difference between R2 and R1 was used to calculate the spasticity index (SI), providing a quantitative measure of spasticity at the elbow. The assessment of the Tardieu Scale was also recorded, and Kinovea software was used to analyze the recordings and accurately measure the R1 and R2 angles (Figure 3B) [24]. In this case, the improvements induced by pPNS were also evident in the elbow spasticity indices, which decreased from 41.77 ± 6.27 (CI95%: 37.78–45.76) to 21.10 ± 6.4 (CI95%: 17.03–25.17), representing a reduction of 49.49% (Figure 3C). However, a similar pattern emerged, with baseline pre-pPNS spasticity levels remaining relatively unchanged throughout the 6-week period (Figure 3D).

### 4.2. Somatosensory Function

During Sessions 1, 6, and 12, a specialized clinician performed pre- and post-intervention somatosensory evaluations to assess the effects of pPNS both locally on the affected limb and systemically by evaluating the contralateral limb. These evaluations targeted four key regions: the thenar area, the forearm, the biceps brachii, and the trapezius (Figure 1B). Tactile thresholds were measured using Von Frey filaments (BiosebLabInstruments) [25], pressure pain thresholds were evaluated with algometry (ChronoJumpBoscoSystem) [26], and conditioned pain modulation was assessed by the cold-press test on the unaffected side [27].

**Figure 4:**
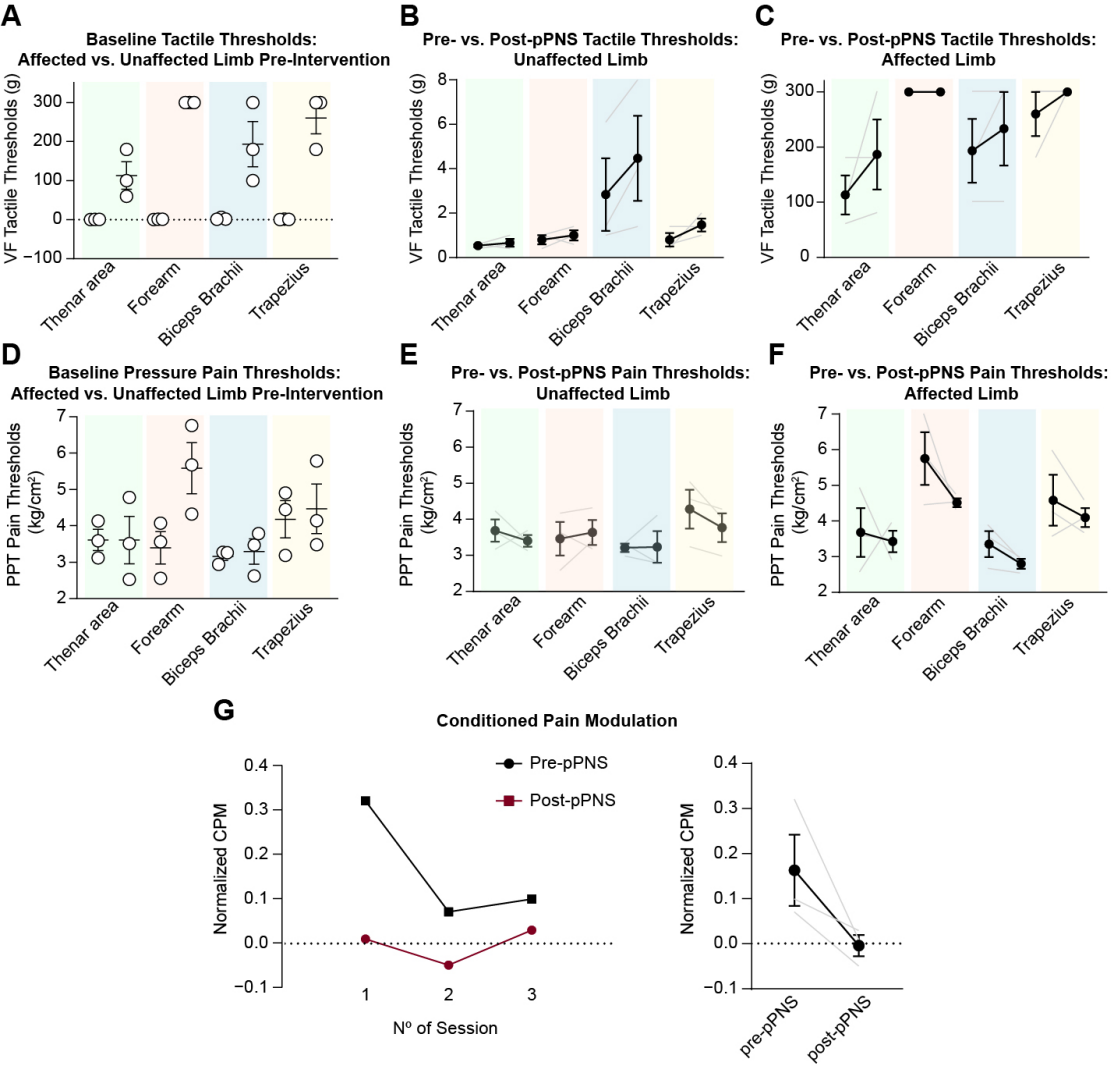
Tactile and pressure pain threshold comparisons, alongside conditioned pain modulation, for the affected and unaffected upper limbs before and after pPNS intervention. (**A**) Baseline tactile thresholds (measured in grams) across various regions of the affected and unaffected limbs prior to intervention. Positive values indicate higher tactile thresholds, suggesting reduced sensitivity, while negative values indicate lower thresholds, suggesting heightened sensitivity. (**B**) Pre- vs. post-pPNS changes in tactile thresholds in the unaffected limb. (**C**) Pre- vs. post-pPNS changes in tactile thresholds in the affected limb. (**D**) Baseline pressure pain thresholds (PPT, kg/cm^2^) for the affected and unaffected limbs before intervention. Higher PPT values indicate lower sensitivity to pressure pain, while lower values reflect higher sensitivity. (**E**) Pre- vs. post-pPNS changes in PPT for the unaffected limb. (**F**) Pre- vs. post-pPNS changes in PPT for the affected limb. (**G**) Conditioned pain modulation (CPM) effects, showing normalized data over three sessions (left panel) and a summary comparison pre- and post-intervention (right panel). Abbreviations: VF, Von Frey filaments; PPT, pressure pain threshold; CPM, conditioned pain modulation; pPNS, percutaneous peripheral nerve stimulation.

Initially, baseline conditions between the hemiplegic and less affected sides were characterized in terms of tactile and pain thresholds. As expected, tactile thresholds were higher on the hemiplegic side compared to the less affected side (thenar area: 0.53 ± 0.11 g vs. 113.3 ± 61.10 g, forearm: 0.8 ± 0.34 g vs. 300 g; biceps brachii: 2.83 ± 2.8 g vs. 193.3 ± 100.7 g; and trapezius: 0.8 ± 0.52 g vs. 260 ± 69.28 g) (Figure 4A). On the other hand, pressure pain thresholds showed a tendency to be higher as well; however, the differences were less pronounced (thenar area: 3.68 ± 0.53 kg/cm^2^ vs. 3.68 ± 1.18 kg/cm^2^, forearm: 3.46 ± 0.80 kg/cm^2^ vs. 5.75 ± 1.27 kg/cm^2^; biceps brachii: 3.21 ± 0.19 kg/cm^2^ vs. 3.35 ± 0.63 kg/cm^2^; and trapezius: 4.28 ± 0.93 kg/cm^2^ vs. 4.58 ± 1.24 kg/cm^2^) (Figure 4D). In both cases, pPNS demonstrated a slight ability to modulate somatosensory function. Tactile thresholds tended to increase in both limbs post-pPNS. On the less affected side, increases of 0.13 g, 0.20 g, 1.63 g, and 0.66 g were observed for the respective areas (thenar area, forearm, biceps, and trapezius) (Figure 4B). On the hemiplegic side, where the intervention was applied, a similar increase was observed: 73.4 g (thenar area), 0 g (forearm), 40 g (biceps brachii), and 40 g (trapezius) (Figure 4C). In contrast to tactile thresholds, pressure pain thresholds decreased after the intervention. In the less affected limb, changes of −0.287 kg/cm^2^, −0.173 kg/cm^2^, +0.023 kg/cm^2^, and −0.513 kg/cm^2^ were observed in the corresponding areas (thenar, forearm, biceps, and trapezius) (Figure 4E). Similarly, in the hemiplegic limb, decreases of −0.253 kg/cm^2^ (thenar), −1.24 kg/cm^2^ (forearm), −0.553 kg/cm^2^ (biceps brachii), and −0.486 kg/cm^2^ (trapezius) were observed (Figure 4F).

Paradoxically, these changes could potentially be attributed to an alteration in the descending pathways. Conditioned pain modulation (CPM) refers to a natural mechanism in which the perception of pain is modulated (either decreased or increased) by applying a conditioning stimulus. In this study, CPM was assessed using the cold-press test [28], where a painful cold stimulus serves as the conditioning stimulus, and the effect on pain perception in another region of the body is measured. Typically, CPM reflects the ability of the central nervous system to inhibit pain when a competing painful stimulus is present. Interestingly, our data indicate that pPNS tended to reduce CPM in our subject (Figure 4G). Conditioned pain modulation decreased from 0.16 ± 0.13 pre-intervention to 0.03 ± 0.007 post-intervention, reflecting an 81.25% reduction from baseline levels. Thus, the reduction in pain thresholds may align with a decrease in conditioned pain modulation.

### 4.3. Tolerability, Subjective Patient Reports, Safety, and Intervention Adherence

In general, the intervention demonstrated a favorable safety profile, with only mild, sporadic, and short-lived adverse effects. These included minor discomfort during pPNS application, which resolved immediately after stopping electrical stimulation. In particular, significant bruising appeared around the biceps brachii area in mid-study (session 6). However, this may be attributable to a congenital disease associated with prothrombin deficiency, as the subject reported a predisposition to easy bruising in other areas without identifiable external causes. This factor should perhaps be considered when the intervention is administered frequently.

Overall, the treatment was well tolerated. The intensity of pPNS was adjusted according to subjective perceptions of the subject daily and the tolerated amperage remained consistent throughout the study, indicating that neither frequency nor dose needed to be increased over time, suggesting no apparent development of tolerance. At the subjective level, multiple nonquantifiable effects and findings were reported (Table 1). Most of these effects were consistently elicited in 100% of the sessions, indicating that they were not incidental but rather contingent on pPNS application.

Finally, it should be noted that this therapy is time-consuming. Nonetheless, whether due to peripheral stimulation, needle puncture, the clinical context, or associated psychosocial factors, the patient demonstrated good adherence to therapy. There were no missed sessions, and the patient expressed a desire for long-term exposure to this therapy, noting favorable effects compared to botulinum toxin.

## 5. Discussion

The treatment of spasticity is a controversial topic [29], leading to questions about the true motivation behind it. Despite much of the literature, including this article, striving to demonstrate treatments to reduce it, some authors and clinicians argue that PSS could be a ’functional adaptation’ of the pyramidal tract in response to injury [29]. Depending on its presentation, it can even provide advantages for patient functionality. From Sherrington’s discoveries, it was observed that this context involves the dysfunction of stretch reflexes, with increased activity in muscle spindles combined with the disruption of neural communication, resulting in net disinhibition [3,30]. However, there is also a non-neural component of spasticity due to sustained muscle contraction, which could cause structural changes in tissue over time [31]. Beyond this, the controversial aspect arises when PSS, despite its potential adaptive component, becomes a factor linked to complications [1,3]. These include post-stroke pain, interference with positioning, mobility, comfort, and hygiene, among others [3]. Therefore, addressing PSS should not be seen as a dichotomous decision, but rather as a gradient to be managed as needed.

In this case report, the patient’s spasticity was identified as a condition affecting their well-being, and managing its degree (rather than seeking complete elimination) resulted in significant improvements in their quality of life. Despite this, several pharmacological and non-pharmacological approaches proved unsuccessful, leading to the reliance on frequent conservative treatments, primarily physical therapy. With the intervention of pPNS, significant reductions in PSS were observed, along with a range of improvements reported by the patient. Both electrical stimulation and needling have shown potential effects [9,32,33,34]. Unfortunately, the limitations of this study prevent a comparison of pPNS with more established approaches such as TENS or NMES. However, it is plausible to assume that the mechanisms of action are relatively shared. The main issue lies in the fact that the mechanisms that mediate the reduction in spasticity remain unknown. Although neuroimaging techniques have been used in stroke patients to study cortical changes in response to peripheral electrical stimulation [35], questions about the spinal mechanisms that mediate the modulation of the stretch reflex remain unanswered. However, changes in myoelectric activity have been observed in the absence of muscle hypertrophy induced by NMES [36,37], suggesting plastic changes and neural adaptations to stimulation. Therefore, spinal plasticity induced by pPNS as a mechanism to reduce spasticity remains a hypothesis that has yet to be confirmed [10].

Considering the somatosensory effects, a paradoxical scenario arises. Certain pPNS interventions may have the capacity to transiently modulate tactile thresholds under healthy conditions [18]. In our case report, pPNS tended to increase these thresholds, suggesting that stroke had not disrupted the pathways responsible for this change (although the validity of this finding is compromised). However, the most intriguing results pertain to alterations in PPTs. Previous evidence shows that electrical stimulation can elevate PPTs, potentially leading to hypoalgesia in pain contexts [38,39]. The mechanisms underlying this effect are complex and cannot be definitively described in clinical studies, although basic research proposes several plausible pathways [40]. In contrast, we observed a trend toward decreased PPTs, in opposition to the tendencies in tactile thresholds. These findings suggest that the intervention effects that mediate the reported changes may operate through different modulatory pathways, one preserved in our stroke patient and another likely compromised. However, valid conclusions cannot be drawn.

Variables such as conditioned pain modulation (CPM) present alterations in various physiopathological conditions [27,41] and may have predictive value in the success of treatment in different pain contexts [42,43]. CPM has been associated with the efficiency of endogenous pain modulation mechanisms, which have inhibitory and facilitatory effects [44]. In stroke patients, pain modulation may be compromised [45]. Some studies do not report significant alterations in CPM [46], while others indicate potential impairments in this modulatory process [47]. In this regard, we observed that pPNS tends to reduce the CPM of our case in alignment with the increased PPTs. This characterization, in our view, is intriguing, as it suggests a possible interaction that may be disrupted in stroke patients with a dysfunctional CPM response, yielding a net facilitatory effect of the intervention. It has also been reported that, in patients with certain brain lesions, CPM depends on the location where the conditioned stimulus is applied [48]. In our study, we measured the systemic effect of CPM exclusively on the unaffected side. It would also have been interesting to assess the potential differential effect on the affected side. Although the intervention demonstrated an overall facilitatory effect, it did not exacerbate pain. However, one could hypothesize that in cases where post-stroke pain is present, certain pPNS protocols might contribute to an intensified pain experience after therapy. Another important question arises from the latter: Are these observed effects dependent on the specific stimulation protocol used? Evidence shows a potential heterogeneity of effects based on the type of intervention [18]. In this case, it is likely that adjusting application protocols or targeting different nerves could allow effective spasticity management without compromising CPM.

Regarding the patient’s point of view, a positive subjective experience was reported, where the patient felt the affected limb was relieved, more functional, less limiting, and, indirectly, important aspects of quality of life, such as sleep and fatigue, were reported to improve, especially on the days of the intervention. In fact, at the end of the 6-week intervention, the patient explicitly expressed a desire to continue with this therapeutic approach, stating that it had been much more effective than botulinum toxin injections. Thus, our preliminary findings suggest the applicability of pPNS to the treatment of spasticity. However, this is a study with no external validity; the conclusions are limited not only by the small sample size but also by the lack of a placebo intervention and potential treatment comparisons.

## 6. Conclusions

This case study presents preliminary evidence suggesting the ability of ultrasound-guided percutaneous stimulation of upper extremity nerves to reduce short-term spasticity, with a favorable personal experience in a refractory case of botulinum toxin. Furthermore, a potential short-term reduction in PPT and CPM is identified, which could be controversial in pain-related contexts where endogenous modulation is compromised. It would be highly valuable not only to investigate these phenomena in a randomized clinical trial with a larger sample and a placebo group but also to contextualize the electrical stimulation protocols used, as the observed effects may vary depending on the intervention.

## Figures and Tables

**Table 1:** Reported pPNS effects.

Reported pPNS Effects	Description	N° of Sessions (%)
Newly developed sensation of limb ownership	Transient increased awareness or sense of control over the limb, as if it is more “integrated” into body image post-pPNS	4 (33.3%)
Arm release experience	Perception of release in the arm, potentially enhancing mobility or reducing perceived tension	12 (100%)
Increase in quality sleep	Improvement in sleep quality reported by the patient, with fewer interruptions and deeper rest	11 (91.67%)
Tingling sensations	Persisting tingling sensation occurring post-treatment and, predominantly, at night	1 (8.3%)
Muscle spasms	Spasms occurring after treatment, causing mild discomfort	3 (25%)
Post-treatment hematoma	Bruising observed at the treatment site following the procedure	1 (8.3%)
Painful muscle contractions	Intense muscle contractions occurring only during the treatment, causing discomfort and requiring adjustment of either (1) the current intensity or (2) the needle positioning to improve patient comfort	6 (50%)
Innocuous muscle contractions	Mild, non-painful muscle contractions that occur only during the intervention. These contractions are noticeable but do not interfere with patient comfort, and no adjustments are typically needed.	12 (100%)

## Data Availability

Data supporting the reported results are not publicly available due to ethical and privacy restrictions associated with the retrospective nature of the study. The data consist of anonymized medical records and are in the possession of the corresponding authors. Access to these data may be granted upon reasonable request and with the approval of the Research Ethics Committee (CER-URL) at Ramon Llull University.

## Abbreviations

The following abbreviations are used in this manuscript:

CPM	Conditioned Pain Modulation
EMG	Electromyography
MAS	Modified Ashworth Scale
NMES	Neuromuscular Electrical Stimulation
pPNS	Percutaneous Peripheral Nerve Stimulation
PPT	Pressure Pain Threshold
PPS	Post-Stroke Spasticity
R1	Passive Range of Motion Angle
R2	“Catch” Angle for Spasticity
ROM	Range of Motion
TENS	Transcutaneous Electrical Nerve Stimulation
TS	Tardieu Scale
